# Feasibility of an altruistic sperm donation program in Canada: results from a population-based model

**DOI:** 10.1186/s12978-016-0275-0

**Published:** 2017-01-14

**Authors:** Daria O’Reilly, James M. Bowen, Kuhan Perampaladas, Riaz Qureshi, Feng Xie, Edward Hughes

**Affiliations:** 1 Department of Clinical Epidemiology & Biostatistics, Faculty of Health Sciences, McMaster University, Hamilton, ON Canada L8S 4 K1; 2 Programs for Assessment of Technology in Health (PATH) Research Institute, St. Joseph’s Healthcare Hamilton, Hamilton, ON Canada L8P 1H1; 3 Toronto Health Economics and Technology Assessment Collaborative, Leslie Dan Pharmacy Building, University of Toronto, Toronto, ON Canada M5S 3 M2; 4 Masters of Science student of Epidemiology and Biostatistics at the University of Western Ontario, London, ON Canada N6A 3 K7; 5 Department of Obstetrics & Gynecology, Faculty of Health Sciences, McMaster University, Hamilton, ON Canada L8N 3Z5

**Keywords:** Models, Reproductive techniques, Spermatozoa, Altruistic sperm donation, Artificial insemination

## Abstract

**Background:**

Stringent donor-screening criteria and legislation prohibiting payment for donor gametes have contributed to the radical decline of donor insemination (DI) using sperm provided by Canadian men. Thus, many individuals rely on imported sperm. This paper examines the feasibility of an altruistic sperm donation (ASD) program to meet the needs of Canadians.

**Methods:**

Using Canadian census data, published literature and expert opinions, two population-based, top-down mathematical models were developed to estimate the supply and demand for donor sperm and the feasibility of an ASD program.

**Results:**

It was estimated that 63 donors would pass Canadian screening criteria, which would provide 1,575 donations. The demand for DI by women was 7,866 samples (4,319 same sex couples, 1,287 single women and 2,260 heterosexual couples).

**Conclusion:**

Considerable effort would be necessary to create the required increase in awareness of the program and change in societal behaviour towards sperm donation for an ASD program to be feasible in Canada.

## Plain english summary

Since 2000, there has been a marked decline in the supply of donor sperm provided by Canadian men. This decline coincides with the introduction of new, more stringent regulations for donor screening and sample testing in 2000, and the proclamation of a criminal law prohibiting payment for donor gametes in 2004. Despite this law, and with limited altruistic alternatives, the importation of sperm from paid donors in the US and Europe has been sanctioned by Health Canada. Since an altruistic donation program is required under the new legislation, the current study was designed to model the number of altruistic donors would be required to satisfy the demand for donor sperm in Canada.

Two population-based, top-down mathematical models were developed to estimate both the supply and demand for donor sperm. “Supply” factors included: population (age and location), ethnic origin, health policy criteria, donor behaviour, and medical eligibility. “Demand” factors included: estimates of utilization by single women, lesbian couples and heterosexual couples.

The evidence-based analysis suggested that only 63 donors would pass Canadian screening criteria, which would provide 1,575 donations. However, the demand for donor insemination was estimated to be 7,866 samples. This results in a shortage of 6,291 donations or 252 donors. These analyses suggested that sperm supplied by donors from other countries is still necessary. The assumptions used in these models suggested that an altruistic program was unlikely to be sustainable unless significant changes occur in the awareness and societal behavior towards sperm donation.

## Background

Using sperm from a third party to overcome infertility is an important option for many women. For some heterosexual couples and most single and lesbian women seeking to conceive, sperm from a fertile, healthy and carefully screened “donor” male remains the most appropriate choice. In particular, for women without a male partner, donor insemination (DI) provided through medical means is the safest and most effective option available. As a reflection of this, single and lesbian women now generate the largest demand for DI [1].

Until 2000, sperm samples from Canadian donors were widely available for use by Canadian women. However, over the last decade the recruitment of Canadian donors has dramatically declined due to the closing of many sperm banks [2, 3]. It has been estimated that the number of Canadian sperm donors was approximately 85–100 before 2000 and approximately 30–50 after this time [4]. Two important events have taken place that explain these closures. The first was the intensification of donor-screening regulations in 2000 [5]. Namely, in July 2000, Health Canada released the Health Canada Directive entitled Technical Requirements for Therapeutic Donor Insemination [5]. These requirements expanded the donor selection criteria and the testing requirements for transmissible diseases. This report stipulated that all prospective donors must be between 18 and 39 years of age with no known hereditary or genetic disease or any serious disability and must have good quality sperm. They must all complete an extensive questionnaire, pass a physical examination, satisfy the rigorous standards for semen analysis, have a risk-free medical and family history, and undergo infectious disease and genetic testing [5]. Some sperm banks may impose other criteria such as a minimum educational background, height, height-to-weight ratio and overall attractive appearance. The second event was the passing into Canadian law of the *Assisted Human Reproduction Act* in 2004 which prohibited “payment for gametes” [6]. With these legal requirements, Canada now has the most stringent requirements worldwide for the screening and testing of semen donors. Whatever the reasons for the trend, currently, it appears that only two sperm banks offer sperm from approximately 60 “altruistic” Canadian donors [7]. Canadian women currently seeking DI thus have to rely on the importation of sperm from other countries. Health Canada permits the importation of “paid donor sperm” with the United States and Denmark being the two main national sources. Sperm from approximately 200 donors are available for importation. This number is small relative to the total number of sperm donors in these countries and is likely related to the stringent criteria as well as the additional costs of compliance with the Health Canada Directive on screening of donors and their sperm [5]. For example, the United States Food and Drug Administration does not require US sperm banks to screen each ejaculate for Chlamydia but this level of testing is required in Canada. US sperm banks therefore only make a limited number of their donors available for export and recover their costs from recipients. The number of donor inseminations from other sources, such as cross border reproductive care or the use of known “fresh” donors, is unknown.

The alternative to the status quo of importing sperm from paid donors into Canada is an effective altruistic sperm donation program, similar to the system in France [8, 9]. This would service the needs of Canadians without contravening the legislated ban on payment for gametes. With just over 4 million men aged between 21 and 40 in Canada, is an altruistic sperm donation program feasible here? This paper seeks to examine the feasibility of an altruistic sperm donor program to service the needs of Canadian women requiring donor sperm.

## Methods

Two models were developed using Microsoft Excel® (version 2010). The first model was designed to estimate donor supply and the other to estimate the demand for donor insemination in Canada. In order to populate the models, a targeted literature search of the electronic databases MEDLINE and EMBASE from 1980 onwards was conducted to identify studies containing information specifically addressing factors that affect sperm supply (e.g., national policy, individual’s behaviour toward altruistic sperm donation (awareness, and willingness to donate), medical eligibility criteria) and demand for donor insemination (e.g., infertility rates, population of same sex couples). These factors were applied to the number of people in the population of interest in Canada based on 2011 Canadian Census data [10] in order to calculate population estimates.

### Model #1: factors affecting altruistic sperm donor population estimates in Canada

A top-down approach was used to estimate the number of potential altruistic sperm donors in Canada. Each step in the model reflects an element that would affect donor supply. The factors included were: population, donor behaviour, medical eligibility and health policy criteria. Age and gender criteria were applied to the population to identify all possible donors in Canada, while behavioural factors, medical eligibility and health policy criteria were used to “filter” the potential pool of donors to estimate the potential altruistic supply of donor sperm.

### Donor population estimate

The Canadian Assisted Human Reproduction Act [6] and the Health Canada directive [5] stipulate that sperm donors must be aged between 18 and 40 years. However, the Canadian Standards Association recommends a minimum age for donors of 21 years [11]. Thus, the total number of males aged 21 to 40 years was obtained from the 2011 Canadian Census of Population (*n* = 4,307,710) [10]. (Table 1).

**Table 1:** Factors Affecting Altruistic Sperm Donor Supply Estimates in Canada

Model Parameters	Base-case	Best-case	Worst-case
Male population base (21–40 years of age)	4,307,710	4,307,710	4,307,710
Proportion of men aware of program	25%	55%	25%
Proportion of men willing to donate	15%	50%	15%
Proportion of men presenting for screening	3%	25%	3%
Proportion of men that pass overall medical screening process	1.3%	1.3%	1.3%
Number of allowable live births	25	25	25

### Donor behaviour

The number of sperm donors in Canada is influenced by factors such as the awareness of a sperm donation program, willingness to donate, and those actually presenting themselves for screening and passing medical screening. One study was identified from the literature review that indicated that 55% of the general male population was aware of an altruistic sperm donation program [12]. It was felt that this was perhaps a bit high for the Canadian context and thus a conservative estimate of 25% was used in the model based on expert opinion [13]. The estimate regarding male willingness to altruistically donate sperm was set to 15% based on the upper limit of studies conducted in the UK, New Zealand and Sweden [12, 14–16]. The percentage of Canadian men actually presenting for screening was set to 3% based on the work of Purdie [12] that showed that only one out of twenty (5%) individuals who said they were willing to donate, actually donated. This value was reduced to 3% following consultation with a Canadian fertility expert based on experience accessing Canadian donor sperm supply (Table 1).

### Sperm donor medical eligibility

The medical eligibility of donors, based on the current screening criteria in place in Canada, was incorporated into the model at 1.3% [17]. Del Valle [17] applied Canadian criteria in one clinic setting, 1/78 or 1.3% of men of eligible age (21–40 years of age) that presented for screening were eligible to be donors.

### Health policy criteria

In an attempt to reduce the risk of accidental consanguinity between donor and conceived people, most countries have implemented reproductive laws related to sperm donation by limiting how many children one single sperm donor may give rise to. In Canada, there is no upper limit to the number of donor offspring. However, a ‘health policy criteria’ filter was incorporated into the model so that the hypothetical impact of employing a policy could be evaluated. The number of live births per donor was set at 25 following US guidance [18].

### Model #2: factors affecting altruistically donated sperm demand population estimates in Canada

The demand for donor insemination was considered to be affected by three factors: the total population of women between 20 and 44 years of age who could potentially want donor sperm (i.e., same sex female couples; single women; and heterosexual couples with infertile men); actual demand for donor sperm from each group; and medical eligibility criteria. For each group, a demand estimate for women/couples seeking sperm was obtained.

### Population estimate for women seeking donated sperm

The 2006 and 2011 Canadian Census data were used to obtain population estimates for each of the three groups (same sex female couples, single women and heterosexual couples) that may be seeking donated sperm. [10, 19] Based on the current clinical screening criteria, it was assumed that women between the ages of 20–44 were eligible for donor insemination (*n* = 5,567,965) (Table 2).

**Table 2:** Factors Affecting Altruistic Sperm Donor Demand Estimates in Canada

Model Parameters	Base-case	Best-case	Worst-case
Female population base (20–44 years of age)	5,567,965	5,567,965	5,567,965
Demand from same sex couples			
Proportion of women living in same-sex relationship	0.528%	0.528%	0.528%
Proportion of female same-sex couples wishing to have children	15%	15%	25%
Proportion of female same-sex couples meeting screening criteria for donor insemination	98%	98%	98%
Demand from single women			
Proportion of single women aged 20–44 years of age	39.3%	39.3%	39.3%
Proportion of single women seeking sperm donation	0.06%	0.06%	1%
Proportion of single women meeting screening criteria for donor insemination	98%	98%	98%
Demand from heterosexual couples			
Number of women in heterosexual relationships between ages 20–44 years	3,335,815	3,335,815	3,335,815
Proportion of couples experiencing fertility issues	16%	16%	16%
Proportion of couples affected by male infertility	24.0%	24.0%	24.0%
Proportion of couples seeking donor insemination	1.8%	1.8%	3%
Proportion of couples meeting screening criteria for donor insemination	98.0%	98.0%	98.0%

### Demand for donor insemination

Canadian data on the demand for donor insemination was not available but a review of the literature identified a Belgian study, which included a large cohort of women (*n* = 1,654) receiving donor sperm between 2000 and 2005 at a single clinic [20]. The requests for donor sperm were reported to be from three groups: lesbian couples, single women and heterosexual couples; with the ratio of use by each group being 5:2:2, respectively. For the purposes of this study it was assumed that the demand in Canada would be similar. It was assumed that the proportion of lesbian couples with children could be used as a surrogate for the number of lesbian couples seeking donor sperm and the 2011 Canadian Census indicated that 15% of the approximately 30,000 same-sex female couples across the country have children [10].

Requests for donor sperm from single women (i.e., not currently married or in a common law relationship, currently separated, divorced or widowed) between the ages of 20–44 was calculated to be 0.06% to provide a 5:2 ratio between the lesbian and single demand (Table 2).

It is estimated that 16% of married and common-law couples experience infertility [21] Of these infertility cases, 30% are attributed to male infertility [22]. Using the 5:2 ratio, the estimated demand for donor insemination by heterosexual couples would be 1.8% (Table 2). It should be noted that the vast majority of heterosexual couples with male infertility now choose other options including intrauterine insemination with the husband’s sperm or, most commonly, in vitro fertilization with intracytoplasmic sperm injection [13].

### Female medical eligibility

The model assumed that of all potential women requesting donor sperm, 98% will meet the eligibility criteria (e.g., no sexually transmitted diseases) for being a suitable recipient (Table 2) [13, 23].

### Base case analysis

The base case analysis employed the best available data to estimate the number of sperm donors and the demand for donor insemination in Canada. The analysis included all men in Canada aged 21–40 years of age (*n* = 4,307,710). It was assumed that 25% of these men would be aware of the need for altruistic sperm donors. Of those, 15% of men would be willing to donate their sperm and 3% would present themselves for all necessary screenings and sample collections. The model assumed that 1.3% percent of men would pass the medical eligibility criteria and 25 allowable live births (Table 1). The demands for donor insemination from same-sex female couples, single women, and married or common law heterosexual couples were set to 15 , 0.06 , and 1.8%, respectively (Table 2).

### Best-case analysis

The best-case scenario regarding donor supply and demand was examined by setting the variables associated with donor behaviour to their most liberal estimates. Of the total male population, 55% would be aware of the need for altruistic sperm donors; of those, 50% would be willing to donate, 25% of those would present themselves for all screenings, 1.3% would pass the screening tests, while the total allowable number of live births remained the same (i.e., 25) (Table 1). From a demand perspective, none of the variables changed from the standard model analysis (Table 2).

### Worst-case analysis

The worst-case scenario was based on status quo estimates for donor variables and liberal estimates for demand. At the same time, the demand for donor insemination was set at its highest levels. It was assumed that 25% of all female same-sex couples, 1% of all single females between the ages of 20–44 and 3% of all heterosexual couples were seeking children through donor insemination (Table 2).

### Estimation of the number of altruistic sperm donors required to meet the demand for donor insemination

The total number of altruistic sperm donors required to meet the estimated demand for donor sperm was calculated by subtracting the total number of donations available for insemination from the number of people requesting donor sperm and dividing this number by the total number of allowable live births per donor (i.e., 25).

## Results

### Base-case analysis

The base-case model predicted that a total of 63 sperm donors (0.0015% of the eligible Canadian male population) providing 1,575 donations would be available (Table 3) while the demand for donations would come from 7,866 women (Table 4) resulting in a deficit of 6,291 donations. Demand is greatest from same sex couples, followed by heterosexual couples, and finally from single women. To balance this model, an additional 252 Canadian donors would be required, assuming a maximum of 25 births from each donor as per US reproductive legislation [18].

**Table 3:** Estimates of Supply for A Hypothetical Altruistic Sperm Donor Program in Canada

	Base case	Best-case	Worst-case
Male population base (21–40 years of age)	4,307,710	4,307,710	4,307,710
Number of men aware of program	1,076,928	2,369,241	1,076,928
Number of men willing to donate	161,539	1,184,620	161,539
Number of men presenting for screening	4,846	296,155	4,846
Number of men that pass overall medical screening process (% of population base)	63 (0.0015%)	3,797 (0.0881%)	63 (0.0015%)
Total number of donations available for insemination (eligible donors permitted 25 live births each)	1,575	94,925	1,575

**Table 4:** Estimates of Demand for A Hypothetical Altruistic Sperm Donor Program in Canada

	Base case	Best-case	Worst-case
Female population base (20–44 years of age)	5,567,965	5,567,965	5,567,965
Number of female same-sex couples eligible for IVF with donor sperm	4,319	4,319	7,198
Number of single women eligible for IVF with donor sperm	1,287	1,287	21,447
Number of heterosexual couples eligible for IVF with donor sperm	2,260	2,260	3,767
Total demand for donor sperm (% of population base)	7,866 (0.1%)	7,866 (0.1%)	32,412 (0.58%)

*IVF* in vitro fertilization

### Best-case analysis

The best-case analysis predicts a total of 3,797 Canadian sperm donors representing 0.0881% of the eligible male population. In this analysis, the demand for donor insemination remained the same as the base-case analysis (Table 4). Using the total number of live births per donor of 25, the supply of donor sperm would exceed the demand by 87,059 and an excess of 3,482 donors (Table 5).

**Table 5:** Estimate of the Supply of Sperm Donors to Meet the Demand for Sperm Donation Insemination

	Base case	Best-case	Worst-case
Difference between donor insemination requests (demand) and donated sperm (supply)	6,291	(87,059)^a^	30,837
Number of additional donors required to meet donor insemination demand	252	(3,482)^a^	1,234

^a^oversupply of donors

### Worst-case scenario

The worst-case scenario, where the availability of sperm donors remains the same as currently available and the donor insemination demand maximized, the total demand by women seeking donor insemination would come from 32,412 women or 0.58% of the eligible population (Table 4). Under these conditions there would be a shortfall in the number of donors of 1,234 in order to meet the demand for donor insemination (Table 5).

## Discussion

This study set out to determine the feasibility of implementing an altruistic gamete donation program in Canada as stipulated in the Assisted Human Reproduction Act [6]. By estimating the potential number of altruistic sperm donors and the demand for donor sperm, it is possible to estimate whether a sufficient pool of altruistic male sperm donors exists to meet the demand for artificial insemination to set future recruitment goals that will satisfy demand in Canada.

The findings indicated that the demand for donor insemination exceeds the potential supply available from eligible and willing Canadian men, providing an estimated donor pool of only 63 men, which is a short fall of 252 Canadian donors to meet the current estimated demand. Currently, the demand is being met by using approximately 250 mostly paid donors from outside of Canada. Further evaluation using a worst-case scenario analysis indicated a significant shortfall in the number of donors. It should be noted that the vast majority of heterosexual couples with male infertility now choose other options including intrauterine insemination with the husband’s sperm or, most commonly, in vitro fertilization with intracytoplasmic sperm injection, which would reduce the demand slightly [13].

The main factors that were considered in the supply model were: population, behaviour variables, medical eligibility, and health policy. The analysis estimated the impact of each on sperm donation in Canada. Only three factors (i.e., health policy, behaviour variables, and medical eligibility) can be modified through changes in health policy or recruitment strategies. The current guidelines regarding health policy put very few social restrictions on the type of men who are eligible to donate sperm, with the only major exceptions being age and being from a high-risk of human immunodeficiency virus (HIV) population (e.g., intravenous drug users). If Canada were to adopt a policy similar to that of France and require donors to be married and have at least one offspring, it would significantly exacerbate the shortage of potential sperm donors. Male behaviour also appears to be a key factor that affects supply: even with the best-case estimates, the potential pool of sperm donors was 0.0881% of the eligible population, with one of the largest barriers to sperm donation being the inconvenience of donating [24]. Del Valle et al. [17] found the recruitment rate for altruistic donors to be less than 1% of those presenting for consideration. Similarly, Paul et al. [25] found that there exists a high level of attrition between presentation and donation, emphasizing the need to maintain a large pool of potential donors. Therefore, looking into strategies that increase male awareness and willingness to donate will most likely ease the shortage of sperm donors.

There have been a variety of methods used in other jurisdictions in order to increase the number of sperm donors, including: 1) asking couples seeking donor insemination to recommend to family and friends the need for sperm donors; 2) asking patients that are about to undergo a vasectomy if they would consider donating sperm; 3) mirror gamete donation, where the partner with usable gametes donates in exchange for the gametes needed (i.e., a man donates sperm in exchange for oocytes and vice versa); and, 4) media campaigns to increase awareness of altruistic sperm donation [25–30]. Whether any of these strategies will work in a Canadian context is yet to be determined and should be introduced and evaluated using an implementation research approach.

Several limitations of this study exist. Firstly, the model assumes that estimates obtained from the literature are reflective of Canadian values and attitudes. Some of the parameters in the analyses were based on studies conducted in other countries. In addition, several parameters, such as Canadian male behaviour towards altruistic sperm donation, require further study. Due to the limited amount of primary research on this topic, all studies that helped identify appropriate variable estimates were used. Furthermore, it was assumed that estimates used for behaviour variables were independent of ethnicity and geography. This may be inaccurate since there is evidence in the literature indicating that certain ethnic groups are more willing to donate than others and there may be differences between people’s propensity to donate from rural rather than urban regions [31–33]. Some of the parameter estimates were obtained from studies that used survey responses. The reliability of these methods to determine actual behaviour may be limited; responses to surveys, especially with respect to organ/tissue donations, may differ from actions. Finally, estimates surrounding demand for donor insemination were based on clinical experience rather than evidence [13]. There were no available data informing the demand for donor insemination from single women, or same sex couples and thus these parameters and other key characteristics of the models were based on a range of assumptions. In addition, the estimate of demand may be overestimated, as women below the age of 25 years and above 40 years of age may be less likely to seek donor insemination. With all of the assumptions made regarding the inputs used, the results of this study are likely to have low generalizability due to differences in reproductive legislation between countries and the reproductive behaviour and attitudes among the populations. However, the model was designed with the capacity and intent to be used to investigate the feasibility of different scenarios using parameters befitting their population (e.g., the feasibility of donor insemination programs if current legislation were to change).

Another factor that was not accounted for in the model was whether having the option of being an anonymous or non-anonymous donor affects the availability of donor sperm. Surveys of sperm donors’ attitudes and motivations toward donor anonymity conducted in Denmark [34] found that 67% of the donors would stop donating if it was no longer possible to assure anonymous donation [34]. Similar concerns arose in Canada when a British Columbia court ruled to end anonymous sperm donation in 2011 [23] but a movement to have the ruling appealed in the Supreme Court of Canada was struck down. Donors in Canada thus still have the right to remain anonymous and ReproMed, Canada’s only nationwide gamete donation centre, has a mixture of both anonymous and non-anonymous donors. Given that donors in Canada have the option to remain anonymous, this factor would not likely have an effect on the availability of donor sperm. However, it is likely that a nation-wide adoption of an open-identification gamete donation policy would decrease the available number of willing donors.

The analysis presented here did not consider the effect of payment on male behaviour towards sperm donation given that payment for sperm donation is illegal in Canada. Evidence suggests that payment does change male willingness to donate, especially among younger donors [34–39]. A study that evaluated the impact of payment for sperm donation found that 32% of donors donated purely for financial reasons [37]. Similarly, Ernst et al. [36] found that 95% of donors were motivated by financial remuneration. When considering donating based on purely altruistic reasons, this number was lower and ranged between 5 and 8% in the above studies [36, 37]. A systematic review published in 2012 found that the primary motives for donating sperm were both altruism and financial compensation [40]. Secondary motive included procreation, passing on their genes and investigating their fertility status [40]. In conclusion, an altruistic sperm donation program could be achieved under optimal conditions in Canada. However, considerable effort would be necessary to create the required increase in awareness of the program and change in societal behaviour towards sperm donation.

## Conclusions

The model created estimated that the potential Canadian donor availability within an altruistic donor sperm program was only 63 donors. Assuming a maximum of 25 live births per donor, only under the best-case scenario with optimized awareness could a Canadian altruistic sperm donation program meet the expected demand. It is expected that the demand will increase due to the increase in funding that provincial governments are infusing into in vitro fertilization.

## Abbreviations

ASD: Altruistic sperm donation
DI: Donor insemination
HIV: Human immunodeficiency virus
